# Edgewise Compressive Properties of Ecological Sandwich Panels with Engineered Bamboo Face Sheets and Bamboo Culm Core

**DOI:** 10.3390/ma18092158

**Published:** 2025-05-07

**Authors:** Xiaoran Liu, Jingjing Deng, Mao Wang, Xinmiao Meng, Lu Xu

**Affiliations:** 1 School of Architecture and Urban Planning, Chongqing Jiaotong University, Chongqing 400074, China; 632106050425@mails.cqjtu.edu.cn; 2 Department of Civil Engineering, Beijing Forestry University, Beijing 100083, China; dengjingjing@bjfu.edu.cn (J.D.); wmwm0695@163.com (M.W.); mengxinmiao@bjfu.edu.cn (X.M.)

**Keywords:** raw bamboo, engineered bamboo, composite sandwich panel, edgewise compression

## Abstract

Bamboo is a green, renewable material with high strength and low cost, but raw bamboo has limited application in residential buildings due to its irregular shape and dry cracking. In this regard, this work proposed a novel ecological sandwich panel to explore the potential combination of engineered bamboo and raw bamboo culms. Face sheets made of glued laminated bamboo panels were bonded to the bamboo culm core via epoxy resin and mortise–tenon joints. Two groups of specimens with height-to-thickness ratios of 4.63 and 5.37 were tested through edgewise compression to investigate the failure modes, strength and rigidity. The results revealed that the specimens had no overall stability problem under axial loading, but exhibited delamination and local bulging to the face sheets. When the height-to-thickness ratio increased from 4.63 to 5.37, but still belonged to the short member range, the area of the adhesive interface increased by 16.13%, and the edgewise compressive strength and rigidity increased by 17.57% and 35.04%, respectively. This indicated that the capacity and rigidity were mainly determined by the connection strength, which was obviously affected by the manufacturing and assembly errors. Accordingly, increasing the connection strength could be helpful for improving the load-carrying capacity and ductility of such panels.

## 1. Introduction

Bamboo, an environmentally friendly and renewable material [1], is widely distributed throughout the world [2]. It grows rapidly and can mature in 3–4 years, and is characterized by light weight and high strength [3]. Its tensile and compressive strengths parallel to the grain are 80–350 MPa and 40–60 MPa, respectively [4,5]. Compared with steel and concrete, bamboo has a high strength-to-weight ratio [6] and shows great potential in building and construction [7]. However, as a natural biomass material, raw bamboo culm is susceptible to ambient humidity changes as well as fungus and insect infestations [7]. When the moisture content decreases below the fiber saturation point, dry shrinkage cracks are likely to occur along the fiber direction [8]. In addition, the irregular shape of raw bamboo makes it difficult to use in residential construction in terms of the requirements for thermal and noise insulation performance. In contrast, engineered bamboo, mainly including bamboo scrimber and glued laminated bamboo (GLB) [9], is characterized by standard sizes and stable mechanical properties, expanding the application of bamboo material. In this regard, it would be reasonable to combine raw bamboo and engineered bamboo for use in low-cost and ecological residential buildings.

Engineered bamboo is manufactured in a similar process to engineered wood, but has better mechanical properties [10,11]. It has been applied to decoration, flooring, furniture and structural elements [12]. However, engineered bamboo has significantly orthorhombic anisotropy, and its strengths parallel to the grain are much better than those in other directions [13,14,15]. Scholars have studied different species of engineered bamboo in compressive tests, showing an average compressive strength of 50–88 MPa [16,17,18]. In particular, among all the engineered bamboo, GLB, also known as laminated veneer bamboo (LVB), draws more attention than scrimber due to its higher strength-to-weight ratio and lower adhesive content [19,20]. It requires a series of treatment processes such as cutting, carbonization and lamination [21,22,23]. Compared with engineered wood, engineered bamboo is a new material but lacks specific test standards and substantial engineering applications [24].

To improve the utilization of bamboo in modern architecture, several exploratory studies have been conducted on bamboo sandwich structures. Tian et al. [25] studied composite columns made of sprayed composite mortar and original bamboo subjected to axial loads. The results revealed that the peak load and ductility of the short composite columns were 1.5 and 2.6 times higher than those of the original bamboo column. Alla Fadlelmola et al. [26] investigated and analyzed the mechanical properties of an engineered bamboo–concrete sandwich panel under axial load, and the influences of different factors including face type, thickness of face and core, and the connection type between the face and the core were discussed as well. The results showed that the bamboo face significantly affected the panel’s mechanical properties. Wang et al. [27] evaluated the bending performance of laminated bamboo sandwich panels with various lattice cores, including triangular, square, and Kagome lattices. The results showed that the laminated bamboo square lattice sandwich panel manufactured using the partition method displayed an excellent bending performance. Darzi et al. [28] proposed a novel sandwich panel manufactured by gluing plywood faces to bamboo core rings through compression and bonding shear tests. The results showed that the ultimate capacity-to-weight ratio of such panels was up to 27.3% higher than that of a conventional cross-laminated timber (CLT) panel of the same dimension under axial compression. At large slenderness ratios, the proposed panels had similar ultimate axial capacities to the CLT panels. In addition, the ductility, stiffness and load-bearing capacity of such panels were positively correlated with the thickness of the panels. González et al. [29] investigated the properties and performance of lightweight sandwich-like composite biopanels made of bamboo, melina and balsa. The results showed that the biopanels were up to two and three times higher than those of solid brick walls and concrete block walls, respectively, and up to almost nine times higher than that of steel in terms of mechanical efficiency. However, current research is mainly focused on the sandwich panels in the combination of engineered timber and bamboo. The feasibility and mechanical performance of novel sandwich panels combining raw and engineered bamboo are yet to be explored.

For a sandwich structure, effective connection at the interface is the key issue in ensuring that the face sheets and the core work together [30]. The connections mainly include metal connection and glue connection, but neither type is able to satisfy all the mechanical demands. Specifically, bolt, screw or nail connectors usually cause large deformations, whereas the glue connection can lead to a brittle failure even though it can achieve higher strength and stiffness [31,32,33]. For the bamboo sandwich structures, the glue connection can be a feasible solution in terms of the low elastic modulus of bamboo [34].

Generally, most studies prefer to use engineered bamboo to form the sandwich structures rather than raw bamboo due to its irregular geometry and susceptibility to cracking, even though raw bamboo offers low cost and sustainability. To explore the potential application of raw bamboo in low-cost and ecological residential buildings, this work proposes a novel sandwich panel composed of engineered bamboo face sheets and bamboo culm core to reduce the impact of external environmental humidity changes on the physical properties of raw bamboo. Furthermore, an axial compression test has been conducted to investigate its mechanical behavior including axial compression strength, ductility, and stability, and to explore the feasibility of using bamboo in the construction of low-cost and ecological buildings.

## 2. Materials and Methods

### 2.1. Material Properties

The test was carried out using side-pressed GLB produced by Hunan Taohuajiang Bamboo Science & Technology Co., Ltd. (Yiyang, China), with a density of 0.69 g/m^3^ and a thickness of 8.5 mm. Moso bamboo (*Phyllostachys edulis*) aged 3–4 years was air seasoned to a moisture content of 12%, and then cut into bamboo culms of 100 mm height. The round bamboo core was selected from the middle segment of the bamboo culm, with an outer diameter of 80–90 mm and no visible defects such as cracks or insect damage. The compressive strength and elastic modulus of the GLB boards were 55.38 MPa and 8.04 GPa, respectively. E51 epoxy resin produced by Shanghai Aotun Chemical Technology Co., Ltd. (Shanghai, China) was used to connect the face sheet and the sandwich core. The ratio of the two-component epoxy resin was 6:1, and the average bonding strength of this epoxy resin was 10.29 MPa obtained via shear tests referring to GB/T 7124-2008 [35]. The ambient temperature of the laboratory was approximately 20 °C with 35–40% relative humidity.

### 2.2. Manufacturing

**Figure 1:**
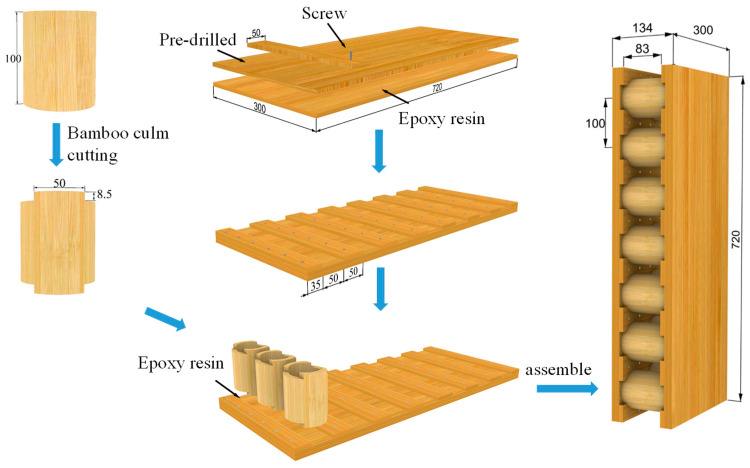
Manufacturing process of the composite sandwich panel.

To provide reliable force transmission between the face sheets and the core, the GLB panels and bamboo culms were connected with glue and mortise–tenon joints. The fabrication process of the composite sandwich panel is shown in Figure 1. Specifically, two GLB panels were first glued together, and then the face sheet was predrilled for the self-tapping screw (M4 × 18 mm). After that, the GLB strips were fixed to the face sheet with screws to form a mortise every 50 mm. The bamboo culm was 100 mm high, with two tenons at a height of 8.5 mm and a width of 50 mm. The face sheets and the bamboo culm core were connected with epoxy resin and pressed for 72 h using G-shaped clamps to ensure a tight junction. The distance between the centers of the bamboo culms was 100 mm. The specimens were divided into two groups with different height to thickness ratios. Details of the specimens are shown in Table 1. The composite sandwich panels could serve as wall panels, so all the specimens were designated as “Wx-y” in Table 1. Due to the limitations of the material and test conditions, only two specimens were repeated for each group.

### 2.3. Setup and Instrumentation

**Figure 2:**
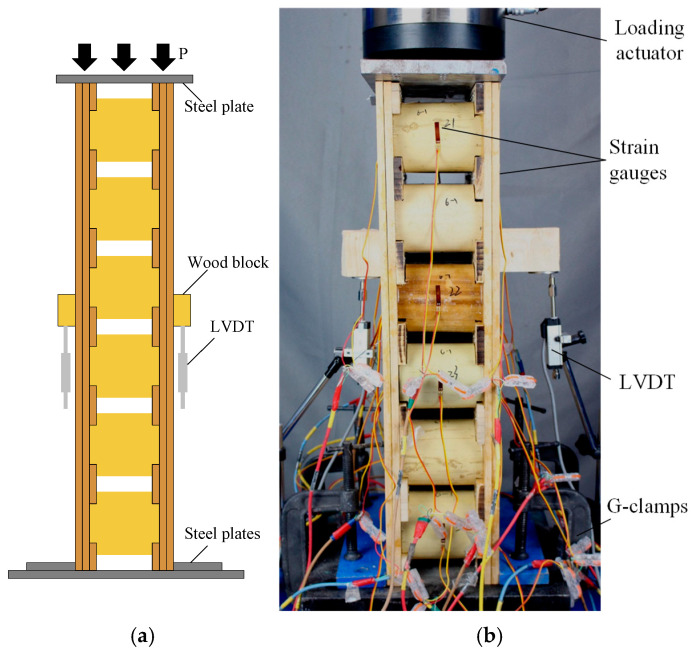
Test setup and instrumentation: (**a**) schematic view; (**b**) setup and measurement.

To investigate the edgewise compressive properties of the sandwich panels, axial compression tests were conducted according to the standard GB/T 1454-2021 [36]. A schematic diagram of the test is shown in Figure 2a. Two linear variable displacement transducers (LVDTs) were placed in the middle of each side of the specimen to monitor the axial displacement of the sandwich panel, and 20-mm-long strain gauges were arranged on the outer surfaces of the face sheets, and on the front and back sides of the bamboo culm core, as shown in Figure 2b. The load was applied at a rate of 4 mm/min during the test until the specimen failed.

## 3. Results

### 3.1. Experimental Observations and Failure Modes

**Figure 3:**
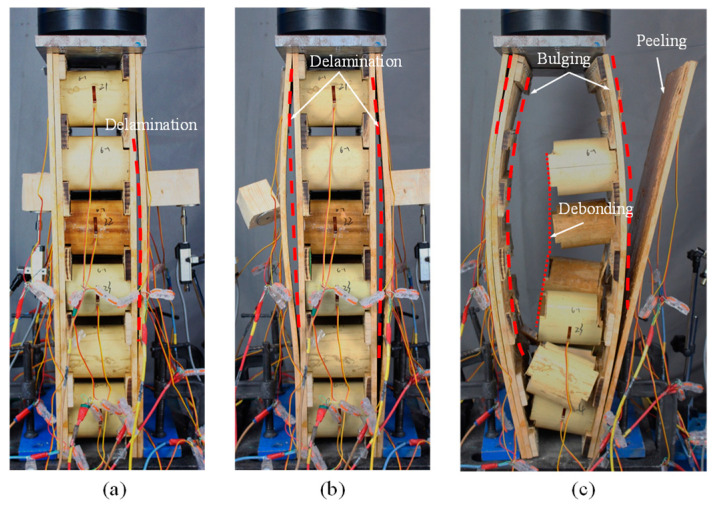
Damage process of W1 specimens: (**a**) single delamination; (**b**) both panels delaminated; (**c**) ultimate failure.

During the test, the outer lamina of the face sheet on one side first partially delaminated at the middle height for group W1, as shown in Figure 3a. Then, both face sheets subsequently experienced delamination failure and lateral flexion, as shown in Figure 3b. As the load increased, the outermost lamina gradually bulged, eventually leading to sudden peeling failure at the joints between the core and the face sheets, as shown in Figure 3c. It could be observed that some bamboo fibers were torn from the face sheets during the peeling process.

**Figure 4:**
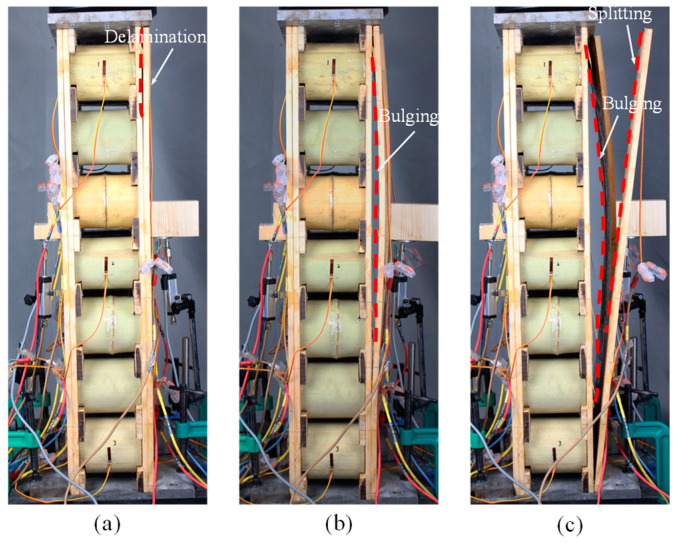
Damage process of W2 specimens: (**a**) laminate delamination; (**b**) panel bulging; (**c**) panel splitting.

Similarly, the outer lamina of the face sheets of the specimen W2 exhibited localized delamination and peeling failure, followed by gradual bulging of the panels, as shown in Figure 4a,b. The damage only occurred on one side of the face sheets. With the increase of the load, the outermost panel of the specimen split along the direction parallel to the grain, as shown in Figure 4c. However, no damage was observed at the interface due to better bonding quality than that of the W1 group.

The failure modes of specimens in the two groups were brittle accompanied by a splitting sound. With the load increasing, the outermost lamina of the specimens was first peeled off from the inner lamina, resulting in a delamination failure of the face sheets. The bonding strength was not sufficient to limit the lateral displacement of the lamina, and brittle damage eventually occurred at the interface for group W1 and at the outermost lamina for group W2, respectively. In reference [27], the sandwich panel was composed of plywood face sheets and a bamboo ring core, which showed global buckling due to the greater length of the specimen. However, the specimens in this work were short components, in which the failure modes resulted from the material damage.

**Figure 5:**
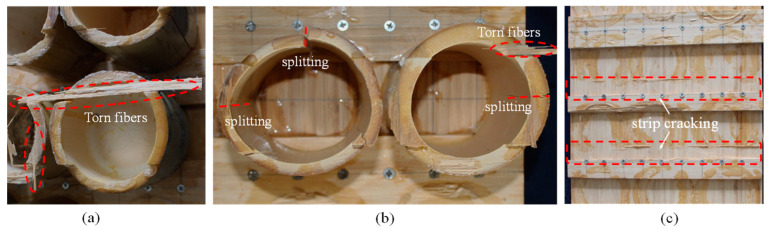
Observation of partial damage to the specimens: (**a**) torn fibers; (**b**) bamboo culm splitting; (**c**) cracked tenons on the face sheets.

A further observation of the cross section of the damaged bamboo culm revealed that some GLB fibers were torn from the laminate, as shown in Figure 5a, which indicated that the bonding strength of the adhesive was greater than the tensile strength perpendicular to the grain of the GLB. Moreover, some bamboo culms exhibited splitting failure in the bamboo wall, which mainly occurred to the bamboo culms without bamboo nodes, as shown in Figure 5b. For the W1 specimens, the GLB strips exhibited splitting failure along the self-taping screw positions due to the weak connection between the GLB strips and the laminate, as shown in Figure 5c.

### 3.2. Axial Load-Displacement Relationships

**Figure 6:**
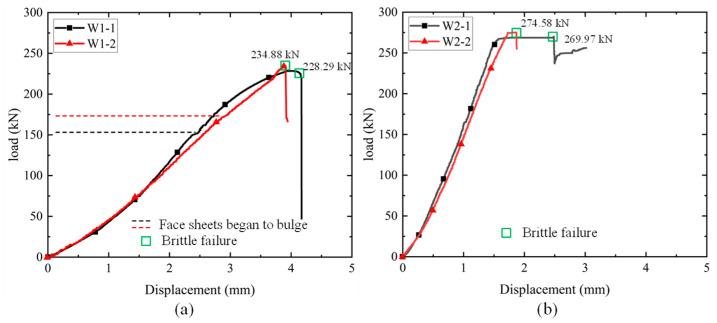
Axial load-displacement curves of the sandwich wallboard: (**a**) W1; (**b**) W2.

The load-displacement curves of the specimens are shown in Figure 6. The initial segment of the curves was obtained by taking the average of the displacement values measured by the LVDTs. However, the face sheets of both groups of specimens bulged as a result of the delamination. The LVDT could not provide exact axial compression. In this regard, the curve segment after the bulging was taken from data recorded via the loading actuator.

The load-displacement curves in each group highly coincided with each other at the load beginning, which could be described as a linear segment. For the W1 specimens, when the outer face sheets of the sandwich panel underwent localized delamination and peeling failure, the slope of the curves started to decrease at approximately 150 kN for specimen W1-1 and 175 kN for specimen W1-2, as denoted by the dashed line in Figure 6a. After that, the stiffness of the sandwich panels gradually decreased until brittle damage occurred. However, for the W2 specimens, the load-displacement curve could be treated as a linear segment before achieving the maximum load as shown in Figure 6b. The maximum load could be kept for a short time when the loading and the local bulging reached a balance, especially for specimen W2-1. Overall, the W1 specimens exhibited typically brittle characteristics upon reaching the peak load in Figure 6a. However, the W2 specimens had a short plateau before the final brittle failure in Figure 6b.

It was found that the W1 specimens had a lower stiffness and load-carrying capacity than that of the W2 specimens, even though the W1 specimens were shorter in height. This was consistent with failure modes. For the W1 specimens, delamination occurred on both face sheets, and bulging was also observed on both sides, which indicated a low bond strength for the laminate. In addition, the interface between the face sheets and the core showed debonding failure. In contrast, the W2 specimens just showed local damage to the face sheet on one side, indicating that fewer initial defects were present in the specimen.

### 3.3. Axial Load–Strain Response

**Figure 7:**
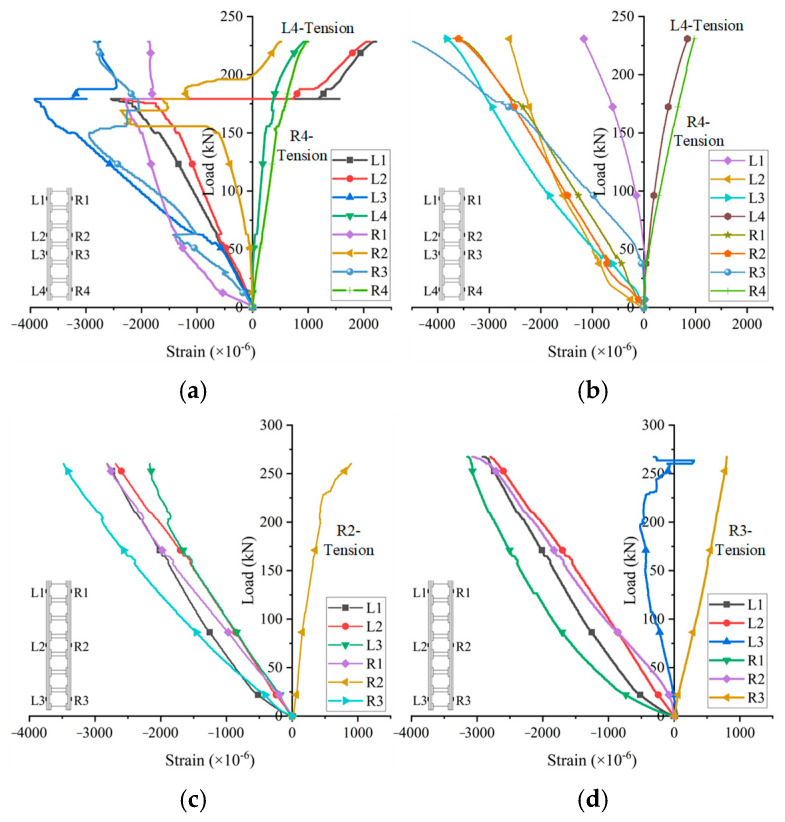
Axial load–strain curves of face sheets: (**a**) W1-1, (**b**) W1-2, (**c**) W2-1; (**d**) W2-2.

The axial load–strain curves of the face sheets are shown in Figure 7, in which the strain values were obtained from strain gauges. In the initial loading stage, all specimens were in the elastic phase and the strain increased linearly. However, when the peak load was reached, the strain changed sharply due to the delamination and the panel bulging.

As shown in Figure 7, the strain values indicated that the face sheets were under compression. Although some exceptive strain gauges exhibited a tensile status, this may have been a result of the local bending of the face sheets. The outward bulging and the subsequent second-order effect would produce local bending. In addition, the delamination and cracking could be deduced by observing the sudden change of the strain value. For specimens W1-1, when reaching 150 kN, the values of strains R2 and R3 had an abrupt change, indicating the delamination of the lamina on right side. When reaching 175 kN, the strains L1, L2 and L3 all showed a sudden value change, indicating the delamination on the left side.

**Figure 8:**
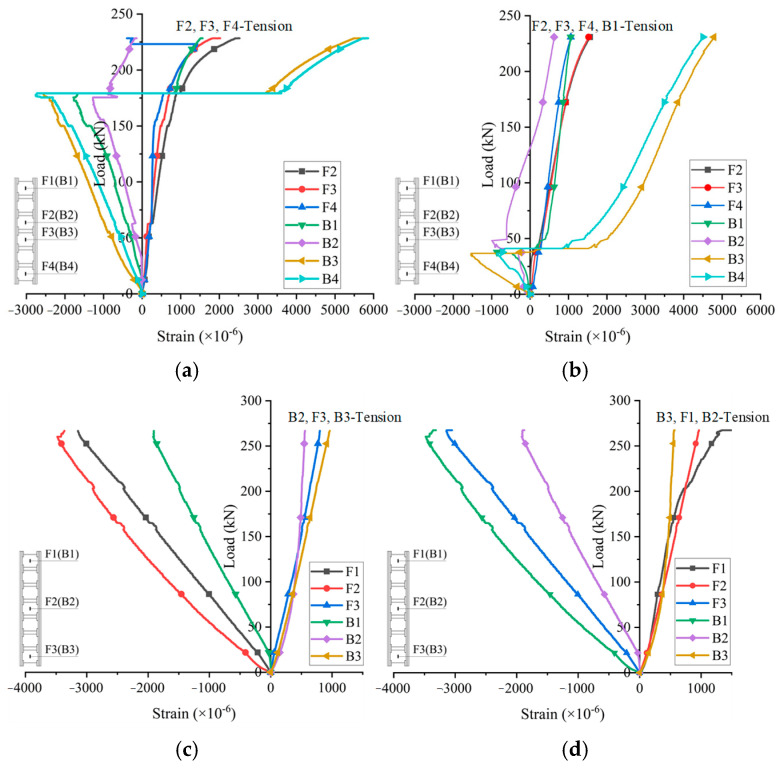
Axial load–strain curves of the bamboo culm core: (**a**) W1-1, (**b**) W1-2, (**c**) W2-1; (**d**) W2-2.

The strain of the bamboo culms included both positive and negative values, as shown in Figure 8, indicating that the outer side of the bamboo culms was under tension or compression in the circumferential direction. Considering the varying diameters of the bamboo culms, the walls were irregularly curved with constantly changing curvatures. The face sheets transferred the load to the bamboo culm through the interface and the mortise–tenon joints. The vertical pressure produced a bending moment on the bamboo wall, so the bamboo wall could be seen as a compression–flexure member. The outer surface of the bamboo culm was subjected to tension or compression on the basis of the relationship between the bending moment and the compression generated by the vertical load. In addition, the strain gauges under tension were not located on one side for most specimens as shown in Figure 8, which resisted the effect of the eccentric loading. However, the error from the cutting and assembling might technically produce strain discreteness.

**Figure 9:**
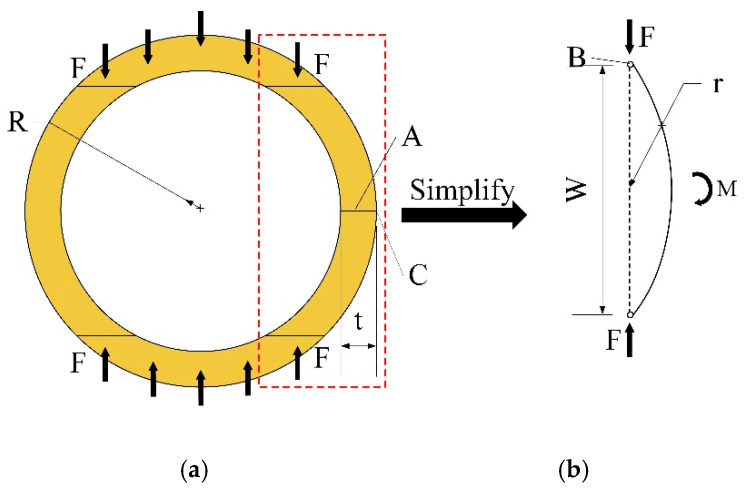
Diagram of the forces on bamboo culms: (**a**) cross section under edge bearing; (**b**) simplified analytical model.

The theoretical analysis of bamboo culms subjected to lateral pressure is shown in Figure 9. The wall of the bamboo culm within the red dashed line in Figure 9a could be simplified to the force model of the press-bent rod shown in Figure 9b according to the force characteristics of the bamboo culm. The simplified bamboo culm was equated to a circular arc with radius r and chord length w.

Taking the detached body AB in Figure 9b, the bending moment generated at the mid-point *A* of the arc under pressure *F* was calculated via Equation (1):

(1) $$M=F\cdot d,d=\sqrt{r^{2}-\frac{w^{2}}{4}}$$

where the *M* is the additional bending moment at the midpoint *A*, *d* is the eccentric distance from the section to the support, and w is the width of the tenon.

The maximum tensile stress $\sigma_{t}$ generated by the additional bending moment at the outermost point *C* could be obtained via Equation (2):

(2) $$\sigma_{t}=\frac{M}{W_{z}},\;W_{z}=\frac{I_{z}}{t/2}=\frac{bt^{2}}{6}$$

where *t* is the wall thickness of the bamboo culm, and *b* is the height of the culm.

Thus, the stress state at *C* under the combined effect of the pressure *F* and the additional bending moment *M* could be calculated as Equation (3):

(3) $$\sigma =\sigma_{t}-\sigma_{c}=\frac{F\cdot \sqrt{r^{2}-w^{2}/4}}{bt^{2}/6}-\frac{F}{\mathrm{bt}}$$

As the raw bamboo was a natural material, the cross-section of the bamboo tube was irregularly circular, so the stress at the outermost point of the bamboo tube was determined by the local curvature and wall thickness.

## 4. Analysis and Discussion

### 4.1. Strength

The load-bearing capacity of sandwich panels with different height-to-thickness ratios could be calculated according to GB/T 1454-2021 [36], and the edgewise compression strength of the panels could be obtained according to Equation (4):

(4) $$\sigma_{f\;}=\frac{P_{max}}{2b\cdot t_{f}}$$

where $\sigma_{f}$ is the edgewise compression strength of the sandwich panel, $P_{max}$ is the peak load, and b and t_f_ are the width and thickness of the face sheets, respectively.

The results are shown in Table 2. The edgewise compressive strength of the composite sandwich panel with a height-to-thickness ratio of 5.37 was 17.57% higher than that of the panel with a height-to-thickness ratio of 4.63. The change in the height-to-thickness ratio was directly related to the size of the contact area of the adhesive layer. No lateral bending, only the bulging of the panels, was observed in either group during the tests. Notably, the compressive strength of the panel was governed by the area of the adhesive layer. A larger adhesive layer would produce a higher load-carrying capacity in the test.

### 4.2. Rigidity

The cross-sectional rigidity *EA* describes the ability to resist deformation under axial loading. The slope of the load–strain curve represented the rigidity of the sandwich panel. To obtain the rigidity, a linear segment of the load–strain curve was chosen for fitting, taking into account the contribution of the panel and the bamboo culm to the rigidity. The results of the linear fitting functions for all specimens are shown in Table 3.

It could be found that the compressive rigidity of the specimens with a height-to-thickness ratio of 5.37 was approximately 35.04% greater than that of specimens with a height-to-thickness ratio of 4.63, demonstrating that the rigidity of the sandwich panel was primarily determined by the adhesive area.

### 4.3. Stability Verification

When the slenderness ratio reaches the critical value of the slender element, Euler’s formula can be applied to calculate its load-carrying capacity.

The slenderness ratio of the specimens was obtained via Equation (5):

(5) $$\lambda =\frac{\mu l}{i},\;i=\sqrt{\frac{I\;}{A\;}}$$

where λ is the slenderness ratio, *i* is the radius of gyration of cross-section, *I* is the second moment of area, and *A* is the cross-sectional area.

The application of Euler’s equation for the calculation of the threshold of the slenderness ratio is shown in Equation (6):

(6) $$\lambda_{p}=\pi \sqrt{\frac{E}{\sigma_{p}}}=\pi \sqrt{\frac{\mathrm{EA}}{\sigma_{p}A}}=\pi \sqrt{\frac{\mathrm{EA}}{P}}$$

where $\sigma_{p}$ is the proportional limitation stress, and *P* is the load corresponding to the proportional limitation. Euler’s formula was applicable to this test if $\lambda >\lambda_{p}$. The specific calculations are shown in Table 4. The results revealed that there was no compression bar stability problem for the specimens and that Euler’s formula was not applicable. In this regard, when the height-to-thickness ratio increased from 4.63 to 5.37, all the specimens belonged to the short member range. The load-carrying capacity and rigidity were mainly determined by material properties and connection quality, which was obviously affected by the manufacturing and assembly errors.

## 5. Conclusions

In this work, a novel ecological sandwich panel was proposed to explore the potential applications of engineered bamboo and raw bamboo culms. Two groups of sandwich panels were tested by edgewise compression tests. The effects of two different height-to-thickness ratios on the compressive strength were investigated. The failure mode was observed, and the compression strength, rigidity and stability were analyzed. The main findings were as follows.

The two groups of panels exhibited different failure modes during the loading process. One of the failure modes was overall bulging of the outermost lamina, whereas the other failure mode was a partial bulging of the outermost lamina. However, the specimens eventually developed with brittle damage at the interface between the face sheets and the core. In brief, the damage to all the specimens was governed by the buckling of the face sheets and the delamination of the adhesive layer.

The face sheets played a dominant role in the edgewise compressive strength and rigidity of the composite sandwich panels. As the height-to-thickness ratio increased from 4.63 to 5.37, the contact area between the panel and the core increased by 16.13%, and the compressive strength and rigidity of the composite panels increased by 17.57% and 35.04%, respectively. These two groups belonged to the short structural member, so the height-to-thickness ratio showed less of an effect on the load-carrying capacity, which was obviously affected by manufacturing and assembly errors.

The results explored the feasibility of the ecological sandwich panel with engineered bamboo face sheets and a raw bamboo core. The failure modes were dominated by the GLB face sheets and epoxy resin, which were industrialized products with stable mechanical performance. Moreover, the raw bamboo culms were placed inside the sandwich panel, avoiding dry cracking due to the environmental impact. In addition, the sandwich core in the wall panel demanded low strength, which also allowed raw bamboo to be produced.

However, owing to the limitations of the material and test conditions, very few specimens were tested in this work. Although the edgewise compressive performance was explored, the errors resulting from the manufacturing, assembly and specimen number could not be ignored. Therefore, the results regarding the mechanical behavior are only indicative, and definitive conclusions can only be drawn based on an extensive experimental program. To promote the application of such panels to the wall component, the adhesive interface and the face sheets needed to be further enhanced. Increasing the connection strength, including the use of a high-quality adhesive and thicker face sheets, would be helpful to improve the load-carrying capacity and ductility of such panels.

## Figures and Tables

**Table 1:** Details of the edgewise compressive test specimens.

Group	Specimen	Size (mm)	Height to Thickness Ratio	Number of Bamboo Culms
A	W1-1	620 × 300 × 134	4.63	18
	W1-2			
B	W2-1	720 × 300 × 134	5.37	21
	W2-2			

**Table 2:** Edgewise compressive strength of sandwich panels.

Specimens	Peak Load *P*_max_ (kN)	Compressive Strength *σ_f_* (MPa)	Average Value (MPa)	Standard Deviation (MPa)
W1-1	228.29	22.38	22.705	0.460
W1-2	234.88	23.03		
W2-1	269.97	26.47	26.695	0.318
W2-2	274.58	26.92		

**Table 3:** Rigidity *EA* of the sandwich panels.

Specimens	Fitting Function	Slope	Rigidity (N)	Average Value (N)	Standard Deviation (N)
W1-1	Linear	0.05577	55.77	59.525	5.310
W1-2		0.06328	63.28		
W2-1		0.08090	80.90	80.385	0.728
W2-2		0.07987	79.87		

**Table 4:** Stability verification of Euler’s formula.

Specimens	Radius of Gyration *i* (mm)	Slenderness Ratio *λ*	Rigidity *EA* (MPa)	*P* (kN)	Threshold Value *λ*_p_
W1-1	58.64	10.57	55.77	228.29	49.10
W1-2			63.28	234.88	51.57
W2-1		12.28	80.90	269.97	54.38
W2-2			79.87	274.58	53.58

## Data Availability

The original contributions presented in this study are included in the article. Further inquiries can be directed to the corresponding author.
